# The status quo of the development of decentralized clinical trials

**DOI:** 10.3389/fmed.2025.1664648

**Published:** 2025-11-19

**Authors:** Xiaoliang Chen, Mingxuan Zhao, Na Lei, Lichun Dong, Qiang Lv, Jiashi Sun, Jianchang He, Xianglin Zhang

**Affiliations:** 1 Shenyang Pharmaceutical University, Shenyang, China; 2 Anning First People's Hospital Affiliated to Kunming University of Science and Technology, Kunming, China; 3 Foundational Medical Faculty of Yunnan University of Traditional Chinese Medicine, Kunming, China; 4 Center for Drug and Medical Device Evaluation of Yunnan Province, Kunming, China; 5 Center for Food and Drug Inspection of Yunnan Province, Kunming, China; 6 The First Affiliated Hospital of Yunnan University of Traditional Chinese Medicine, Kunming, China

**Keywords:** decentralized clinical trials, digital health technology, transformative model, DCTs, digital clinical trials

## Abstract

Decentralized clinical trials (DCTs) have emerged as a transformative model in new drug development, offering alternatives to traditional site-based trials through the integration of digital technologies and remote processes. This literature review examines the current landscape of DCTs by academic studies, policy reports, and regulatory guidance from major global regions. The review identifies and discusses the opportunities and challenges of DCTs, including scientific and operational innovation, equity and accessibility, governance and trust, and sustainability and infrastructure. A comparative analysis of regulatory frameworks and guidance issued by different regulatory authorities all around the world reveals both convergences and distinctions in how DCTs are approached, particularly in areas such as digital health technologies or patient-centered models. The U.S. emphasizes efficiency and technological integration; the EU prioritizes equity and patient engagement; while China focuses on rare diseases, reducing regional disparities, and maintains a more cautious regulatory approach. The review concludes by identifying the need for greater international coordination and harmonization to fully realize the potential of DCTs while addressing their inherent risks. Building on observed regional differences, it further examines the challenges associated with harmonization, the implications of fragmented governance across jurisdictions, and the lessons learned from pilot implementations. These insights aim to inform future efforts toward more cohesive and globally aligned DCT frameworks.

## Introduction

Decentralized clinical trials (DCTs) are an operational model of clinical trials in which some or all trial activities are designed to take place at, or in the vicinity of, the participant’s home, rather than at a traditional clinical site. This approach can make use of technologies and other innovative operational approaches to facilitate data collection (1). In a survey by Oracle (2), findings revealed that as early as the first year of the pandemic, 76% of sponsors and CROs had integrated decentralized elements into their trials. Among these, 7% had fully transitioned to DCTs. As DCTs continue to expand in scope and scale, questions have arisen regarding their practical value, ethical issues, and regulatory oversight. This literature review provides a comprehensive examination of the current status of DCTs, focusing on three sections: (1) the opportunities and challenges of DCTs, which include scientific and operational innovation such as promoting a new paradigm of trial and reducing the burden, equity and accessibility, such as diversity and digital divide, governance and trust, such as cybersecurity and ethical issues, sustainability and infrastructure, such as financial value and ecosystem; (2) the terminology associated with DCTs; and (3) the similarities and differences in regulatory frameworks and elements across major regulatory authorities. By comparing the approaches of regulatory bodies, such as the U. S. Food and Drug Administration (FDA), European Medicines Agency (EMA), and National Medical Products Administration (NMPA), this review aims to explore the points of convergence and divergence that may shape the global future of decentralized clinical research. Ultimately, this study contributes to the broader discussion on how to advance DCTs in a way that is scientifically robust, ethically sound, and globally coordinated.

## Methodology

The latest and current relevant guidelines, regulatory requirements, and available literature about DCTs were systematically searched. Guidelines and regulatory requirements were searched from various institutions in different countries and regions, including the U. S. FDA, Health Canada, European Commission (EC), Heads of Medicines Agencies (HMA), EMA, UK Clinical Research Recovery, Resilience and Growth (RRG) program Board, the Australian Teletrial Program (ATP), International Medical Device Regulators Forum (IDMRF), NMPA, the National Health Commission (NHC), the National Healthcare Security Administration, the State Administration for Market Regulation, and the Cyberspace Administration of China (CAC). Moreover, “decentralized clinical trial,” “digital clinical trials,” “DCTs,” “digital health technology,” “medical devices,” and “medical software” were also used as keywords to search PubMed, Embase, and China National Knowledge Infrastructure (CNKI) database up to September 2025 for relevant clinical studies, reviews, case reports, and other related literature on DCTs. The extracted information mainly involved the DCT and DHT initiatives conducted in different countries and regions, terminology related to DCTs in the different literature and regulatory authorities, key regulatory departments for DCTs, and the elements of DCTs. Any inconsistent results were resolved by discussion between two investigators or reaching a consensus with an additional investigator.

## Promoting a new paradigm of trial

In 2012, the U.S. FDA mentioned a concept named Patient-Focused Drug Development (3). After 4 years, the 21st Century Cures Act also proposed a series of requestion like real-world studies. In this way, clinical trial innovation and transformation have become a trend in recent years.

DCTs facilitate a patient-centered model by actively engaging patients in both the study process and evaluation. Moreover, through the use of digital health technologies, these trials generate more real-world data, which can then be leveraged to create real-world evidence. This, in turn, drives innovation in new drug development.

## Reducing the burden

Participating in clinical trials often requires extra time, affecting work, rest, life, or travel. This may be one reason why many people interested in participating in clinical trials finally decline trials. In addition, investigators also need to spend substantial effort to repeatedly collect some basic health data, which also increases the workload of staff. Decentralized elements and some digital health technologies can effectively reduce the burden on participants and staff (4). Based on the current challenges, they classified these potential elements and technologies into four types: (1) available remote solutions, (2) required additional technical development and/or validation, (3) unlikely to be fully remote, and (4) remote solutions not possible (Table 1).

**Table 1:** Digital solutions in decentralized clinical trials.

Remote solutions available	Additional technical development and/or validation required
Vital signs Biomarker assessment Patient-reported outcome Patient instruction Urinalysis Pregnancy Patient history Continuous glucose monitoring	Clinical chemistry Safety laboratory assessments Respiratory function Pharmacokinetics Clinical assessment Medication usage Adverse event Fraction exhaled NO ICF

Unlikely to be fully remote	Remote solutions not possible
Physical examination Hematology	Central imaging ……

## Diversity and inclusion in clinical trials

Over the past decade, the FDA has been advocating for the diversity of participants in new drug clinical trials. However, the problem of underrepresentation of participants in many clinical trials persists (5). In 2017, the Center for Information and Study on Clinical Research Participation (CISCRP) launched an online questionnaire in 68 countries around the world to learn personal attitudes and experiences toward clinical trials. The questionnaire has been distributed to approximately 120,400 people around the world, but the response rate is only 10%. Moreover, only a small number of people (3,848 people) expressed interest in participating in clinical trials (6). This situation is even more obvious for marginalized groups in society (7). In 2022, the FDA issued a new Reform Act to improve the recruitment and retention of diverse participants in clinical trials, including decentralized elements, digital health technology, and innovative design in recruitment (5, 8).

On the other hand, patients often have a low willingness to participate in clinical trials. This not only affects the diversity of participants but also delays the progress of the clinical trial. In some trials, nearly half of the sites are unable to start due to a lack of participants. As a result, the costs of clinical trials have increased dramatically (9).

## Digital divide

Concerns about the digital divide first arose in the late 1990s, highlighting the disparities in Internet infrastructure between developed and developing countries (10). On 26 March 2024, Christine Bost, President of Bordeaux Metropole, defined the digital divide as a significant gap between individuals who have access to digital resources and those who do not, warning that this divide could worsen existing inequalities and continue to fuel social exclusion throughout Europe (11). Understanding digital technology is like learning how to read. It represents a fundamental way to acquire knowledge and a basic life skill. To bridge the digital divide, it is essential to gain a deep understanding of it, because what cannot be measured cannot be managed (12).

The digital divide, a common public issue, also exists in DCTs. Learning new digital technologies and limited Internet access create barriers, especially for vulnerable groups. Therefore, we need more support from the government and society to close the digital gap, not widen it.

## Cybersecurity and data protection

In 2022, Section 524B was added to the Federal Food, Drug, and Cosmetic Act to strengthen cybersecurity for medical devices (13). In 2023, the FDA issued guidance for industry on cybersecurity considerations and documentation for device premarket submissions. These guidelines are also being applied to digital clinical trials (14).

Additionally, the European Union’s General Data Protection Regulation (GDPR), which came into effect in 2018, provides comprehensive provisions for the protection of personal information. It requires that sponsors safeguard the confidentiality of clinical trial participants’ data, an important consideration in the early design of DCTs. Sponsors should plan activities involving digital health technologies as early as possible to ensure compliance with GDPR guidelines and reduce the risk of data-related noncompliance.

## Review of the ethical and legal framework

Over the past decade, several innovative clinical trial models have gradually emerged. The use of DCTs and digital health tools, such as recruitment, electronic informed consent, telemedicine, collection of patient-reported outcome measurements, and safety monitoring, has introduced a number of ethical challenges. *Effy Vayena and others* (15) divided these ethical issues into three areas that need to be improved: 1. Personal safety and rights, such as self-storage and monitoring investigated products, as well as protecting participants’ privacy in the data era. 2. Scientific validity, alternatives like replacing site-based endpoints in traditional trials with digital endpoints or digital biomarkers must be scientifically validated. 3. Ethical oversight, while decentralized clinical trials and digital health tools can address many limitations of traditional trials, not all trials are suitable for this model. To sum up, implementing decentralized clinical trials, an innovative clinical trial model, also presents the challenge of aligning ethical and legislative standards.

## Barriers to new infrastructure and ecosystem

Decentralized clinical trials rely on good infrastructure and ecosystem, and existing clinical trial infrastructure usually changes slowly, which leads to some challenges and difficulties in the process of transforming traditional clinical trials into a new paradigm of clinical trials. A report *Governing health futures 2030: Growing up in a digital world* from the Lancet and Financial Times Commission recommends investing in digital technology and public health in a national strategy to establish the national digital infrastructure (16). Reliable physical and operational infrastructure, such as power supply, Internet access, telephone, smartphones and apps, email, communication systems, and cold-chain logistics for investigational medicinal products and specimens, is essential. Additionally, software and systems used in clinical trial activities, including digital health tools, must meet basic requirements such as system validation, audit trails, and electronic signatures. Local healthcare systems outside the study institution should also be acceptable. On the regulatory side, authorities need to update existing guidelines. For example, with the rapid development of digital health technologies, the definition of traditional medical devices is expanding and requires redefinition. Furthermore, as these new digital health products and devices collect data, their accuracy and feasibility must be validated. In response, the FDA has launched several initiatives, including the Digital Health Innovation Action Plan and the Software Pre-certification Pilot Program. Another obstacle to improving infrastructure and ecosystems is the lack of standardization and collaboration. In Table 2, we have outlined some innovative clinical trial actions by major global regulatory entities such as the United States, Canada, Europe, and the United Kingdom, and their funded partner (9, 17–28). There is a global agreement and shared vision regarding the potential of digital health technologies; however, effective international collaboration remains limited, as demonstrated by the inconsistency in key concepts and terminology (29).

**Table 2:** Decentralized clinical trial and digital health technology initiative conducted in different countries and regions.

Country/Regions	Organization	Vision/Works
United States	FDA	Digital Health Innovation Action Plan Software Pre-certification Pilot Program A series of new guidance about DCT & DHT.
	Clinical Trials Transformation Initiative (CTTI)	Build digital health trials
	National Institutes of Health (NIH) National Science Foundation (NSF)	Chart a path forward for greater implementation of digital clinical trials
	The Trial Innovation Network is supported by NIH.	Addressing roadblocks in clinical trials and accelerating the translation of novel interventions into life-saving therapies.
	Decentralized Trials and Research Alliance (DTRA)	Enables collaboration between stakeholders to accelerate the adoption of patient-focused, decentralized clinical trials. Makes participation accessible to everyone. Promotes decentralized trials by establishing clear goals and recommendations. Explores the partner organizations that collaborate to drive innovation and progress in the life sciences and clinical trials ecosystem.
Canada	Health Canada	Regulatory review of drugs and devices
Europe	European Commission (EC) Heads of Medicines Agencies (HMA) European Medicines Agency (EMA)	Digital Transformation of Health and Care Recommendation paper on decentralized elements in clinical trials EU Clinical Trials Regulation (EU CTR) to modernize and harmonize the regulatory framework for clinical trials across the European Union.
	Innovative Medicines Initiative (IMI)	Trials@Home, an IMI-funded research project about DCT.
UK	UK Clinical Research Recovery, Resilience and Growth (RRG) program Board.	Patient-centred research Research enabled by data and digital tools
Australia	The Australian Teletrial Program (ATP)	Improves access to and participation in clinical trials for rural, regional, and remote Australians.
Global	International Medical Device Regulators Forum (IDMRF)	In 2013, the first key definition was defined: Software as a Medical Device (SaMD).

## Financial value

A study (30) developed an expected net present value (eNPV) model to evaluate the financial impact of DCTs on new drug development and commercialization cash flows, as well as to calculate the return on investment (ROI) of DCTs. The study found that applying decentralized clinical trials to phase II and phase III trials could increase the ROI of a drug prior to phase II by seven-fold. Another report from Deloitte in 2019 (31) showed that the average cost of launching a new drug increased from US$1.188 billion in 2010 to US$1.981 billion in 2019, while the average predicted maximum sales volume decreased from US$816 million to US$376 million over the same period. As a result, the expected internal rate of return (IRR) dropped from 10.1% in 2010 to 1.8% in 2019. This decline in IRR is attributed to inefficiencies in the current drug discovery and clinical development processes. Over the past decade, the return on investment for new drugs launched via traditional clinical trial models has fallen, whereas the use of decentralized elements in clinical trials has shown benefits in improving development efficiency and reducing time-related costs.

## Terms

Across various scientific publications, think tank reports, expert opinions, and regional regulatory guidelines, there is a noticeable lack of consistency and standardization in the terminology used to describe DCTs. Terms such as “digital clinical trial,” “virtual clinical trial,” “decentralized clinical trial,” and “teletrial” are often used interchangeably or inconsistently, which creates confusion and hinders effective communication among stakeholders. Here, we collected the different terms described in the different scientific literatures and organizations, as shown in Table 3. These terms can be grouped into six main categories. We believe that key terms, such as DCTs and digital health technology, should gradually reach international consensus and be systematically presented in several globally influential and authoritative guidelines. By establishing a clear and well-structured terminology system, stakeholders can avoid confusion and enhance communication when discussing the design, development, and implementation of DCTs (32).

**Table 3:** Terminology related to decentralized clinical trials in the different literature and regulatory authorities.

Subjects	Terms
Trial model and New Paradigm	Virtual Clinical Trials, Internet-based trial, Site-less clinical trial, Online clinical trial, Direct-to-patient clinical trial, Web-based clinical trial, Networked trial, Patient-centric clinical trial, Remote clinical trial, Decentralized clinical trial, Digital clinical trial, Teletrial. Real-world study Patient-focused drug development Fully decentralized clinical trials Hybrid decentralized clinical trials
Principle	Patient-centric Fit-for-purpose Risk-based quality design
Infrastructure	Internet Direct to patient At-home medication delivery Safety monitoring
Digital health technology, DHT	Software as a Medical Device (SaMD) Software in a Medical Device (SiMD) Medical Device Data System (MDDS) Mobile Medical App (MMA) Wireless, Wearable, Sensor Consumer product/Digital public goods Firmware Artificial Intelligence Application programming interfaces (APIs) Context of use (COU) Digital Technology Telehealth Outcome Assessment Structured Data Validation and Verification General-purpose computing platform Usability studies/Usability testing
Electronic data	Clinical outcome assessment, Patient-reported outcome, Data life cycle, Remote data acquisition, Electronic source, DDC/Direct entry, EHR, Non-CRF, Device and Apps, Access Control, Transfer, Transcription, Cloud solution, Migration, Transmit
Outcome	Drug Development Tool (DDT), Medical Device Development Tool (MDDT), Digital endpoints/biomarker, ePRO, eCOA

## Key actions in Europe and the United States

With the development of global digital health technologies and the outbreak of the COVID-19 pandemic, the advancement of DCTs has been significantly accelerated. Regulatory authorities in various religions have formulated digital health action plans and successively issued a series of policy documents to reflect their regulatory expectations and requirements. The major related actions in regions such as the United States, Europe, the United Kingdom, and Canada are shown in Table 2. Meanwhile, the United States mainly proposed two innovative clinical trial paradigms, such as Patient-Focused Drug Development and Real-World Studies. They defined the SaMD from the IMDRF, and advanced digital health initiatives such as the Digital Health Innovation Action Plan and the Software Pre-Certification Pilot Program. They also issued a series of guidelines to build a regulatory framework and guide the ecosystem of innovative clinical trials. A summary of the US FDA’s key actions and timelines is provided in Table 4.

**Table 4:** FDA’s key actions and timeline about DCT and DHT.

Year	Action	Main contents
2012	Food and Drug Administration Safety and Innovation Act (PDUFAV).	Section 1137, Patient Participation in Medical Product Discussions Workshops and Partnerships, including Clinical Trials Transformation Initiative (CTTI) FDA Patient Representative Program Patient-Reported Outcome (PRO) Patient-Focused Drug Development Initiative (PFDD)
2015	Clinical Trials Transformation Initiative (CTTI)	It was co-founded by Duke University and the U. S. Food and Drug Administration in 2007. ‘Real-World Impact’, ‘patient-centered’, ‘making patients equal partners’, and ‘Mobile Technologies’ first occurred in 2015.
2016	21^st^ Century Cures Act	Patient Experience Data Real-world evidence (RWE) Clarifying medical software regulation
2017	Food and Drug Administration Reauthorization Act (PDUFAVI)	PFDD public workshops to inform guidance FDA Reauthorization Act of 2017 DEVICE INSPECTION AND REGULATORY IMPROVEMENTS Device pilot projects (FY2017-2022) Electronic Submissions and Standardization of Electronic Application Data
2017	Digital Health Innovation Action Plan	Issuing new guidance to provide clarity on the medical software provisions of the 21st Century Cures legislation Launching an innovative pilot precertification program Reimagining digital health product oversight; exploring a new streamlined pathway for software. Building the FDA’s bench strength and expertise in CDRH’s digital health unit.
2017	Software Pre-certification Pilot Program	Enables a modern and efficient regulatory framework that allows software iterations and changes to occur in a timely fashion. From Concept to A Program: An Iterative Approach This pilot, limited to manufacturers of software as a medical device (SaMD), is an important first step to help us explore and evaluate the program model to inform how we establish the Pre-Cert Pilot Program.
2022	PDUFA VII	Publishing a Framework for the Use of Digital Health Technologies in Drug and Biological Product Development.
2022	Food and Drug Omnibus Reform Act of 2022	Section 3606 requires the FDA to publish guidance to advance the use of decentralized clinical trials, defined as clinical studies in which some or all of the study-related activities occur at a location separate from the investigator’s location.
2023	Decentralized Clinical Trials for Drugs, Biological Products, and Devices.	Provides recommendations for sponsors, investigators, and other stakeholders regarding the implementation of DCTs for drugs, biological products, and devices.
2023	Digital Health Technologies for Remote Data Acquisition in Clinical Investigations	Uses digital health technologies (DHTs) to acquire data remotely from participants in clinical investigations that evaluate medical products.

## Framework and elements of DCTs

In recent years, regulatory authorities in major regions around the world have actively issued regulatory frameworks and key elements for DCTs, in order to provide guidance to investigators and sponsors. In China, as of mid-2025, regulatory authorities have not yet issued a standalone and comprehensive guideline addressing all forms of DCTs. However, it is possible to summarize relevant regulatory frameworks, expectations, and requirements for digital, software-based, remote, and AI-assisted clinical trials, as well as digital therapeutic products, based on current regulations, guidelines, official announcements, and practices. Several of these practices and regulatory documents address key aspects of DCTs, such as remote monitoring, electronic informed consent, and data management systems. These are mostly governed by various guidelines and laws from Chinese regulatory authorities, including NMPA, the National Health Commission (NHC), the State Administration for Market Regulation (SAMR), and the Cyberspace Administration of China (CAC). The major regulatory departments and their roles are presented in Table 5, along with the key regulations and practices related to these areas, which are presented in Table 6. We compared the regulatory framework and elements for decentralized clinical trials from three different regulatory agencies: the US FDA (33), EMA (24), and NMPA (China) (34). There are several similarities and differences. The similarities are primarily reflected in the following aspects. First, digital economy strategies have been launched at the national level across various fields, driving innovation and new paradigms in drug development. For instance, the 21st Century Cures Act in the United States, the European Digital Health and Care Strategy, and China’s Internet Plus Healthcare initiative have all served as strategic frameworks promoting digital transformation in the field of drug development. Second, the primary regulatory authorities overseeing decentralized clinical trials (DCTs) in each region have issued a series of expectations and requirements. These include decentralized elements such as digital health technologies in the United States, patient education in Europe, and cybersecurity in China. While these components are relevant to DCTs, they also extend beyond the specific scope of DCT frameworks (Tables 2, 4, 6 for details). Third, a review of representative regulatory guidelines from different global regions reveals a relative convergence around the frameworks and core elements of DCT regulation, as shown in Table 7. These can be broadly categorized into five key areas: risk assessment of the new paradigm, study design, digital elements (e.g., eIC, DTP, telemedicine, and remote monitoring), data governance, and stakeholder communication. Fourth, both the United States and Europe have gone beyond regulatory efforts by providing substantial financial support to initiatives that promote innovative research. Notable examples include the Clinical Trials Transformation Initiative (CTTI) in the United States and the Innovative Medicines Initiative (IMI) in Europe. Finally, although the decentralized clinical trial (DCT) paradigm is still in the process of being refined, regulatory frameworks across different regions have unanimously placed strong emphasis on privacy protection and data security. For example, the United States has implemented the Health Insurance Portability and Accountability Act (HIPAA, 1996) along with a series of related FDA guidelines in recent years; the European Union has enacted the General Data Protection Regulation (GDPR); and China has introduced the Data Security Law and the Personal Information Protection Law.

**Table 5:** Key regulatory departments and their roles in China for DCTs.

Agency/Department	Main Role/Responsibilities relevant to DCTs
National Medical Products Administration (NMPA)	Primary regulator for the registration affairs of drugs, medical devices, and cosmetics, with overall oversight of clinical trials, including decentralized clinical trials (DCTs). Responsible for developing Good Clinical Practice (GCP) guidelines and organizing supervision and inspections.
National Health Commission (NHC)	Responsible for the qualification of healthcare institutions and clinical trial sites. Oversees healthcare services, including the management of hospital digitalization, telemedicine and Internet-based services, electronic medical records (EMRs), healthcare infrastructure, and ethical governance. Collaborates with the NMPA on clinical research matters related to personnel qualifications, ethical oversight, and site regulation.
Cyberspace Administration of China (CAC)	This involves medical data, personal privacy protection, cybersecurity, and the hierarchical protection of information system security. Healthcare data is generally subject to the national classified information security protection framework.
State Administration for Market Regulation (SAMR) Other Relevant Bodies.	Responsible for establishing national standards, organizing technical forums, offering policy advice, and participating in the discussion and formulation of guidelines.

**Table 6:** Overview of China’s regulatory framework and practices for DCTs.

The key elements	Regulatory agency	Decentralized or digital aspects	Law, regulation, or guidance
Concept and basic elements of DCTs.	National Medical Products Administration. (NMPA)	Propose electronic data Collection/management, remote monitoring, and virtual visits. Telemedicine	China GCP (2020) ICH-GCP (2025) Guidelines for Clinical Trial Data Management (2016) Guidelines for Electronic Data Collection in Clinical Trials (2016)
Medical devices	National Medical Products Administration. (NMPA) National Health Commission (NHC)	Propose the definition and scope of digital health technologies related to DCTs. Manage them based on risk classification with reference to medical device regulations. Many digital health technologies used in DCTs, such as wearables, diagnostic devices, and remote monitoring systems, are regulated as medical devices.	Good Clinical Practice for Medical Devices (2022)
Telemedicine activity (Infrastructure)	National Health Commission (NHC)	Telemedicine, in-person visits, or a hybrid healthcare system.	Administrative Measures for Internet-based Diagnosis and Treatment (for Trial Implementation, 2018) Administrative Measures for Internet Hospitals (for Trial Implementation, 2018) Supervision Rules for Internet-based Diagnosis and Treatment (for Trial Implementation, 2018).
Medical Device Software, Health IT Systems, and the internet.	State Administration for Market Regulation National Health Commission (NHC)	The use of medical software and health information technologies (such as Electronic Health Records, EHRs) in clinical trials. Digital platforms that collect and manage trial data. Healthcare services include digital health, telemedicine, mobile health (mHealth), and other innovative technologies.	Information security technology-Guide for health data security (2021) Promoting the Development of “Internet Plus” Healthcare (2020)
Ethic requirement	National Health Commission (NHC)	Expand the scope of ethical oversight, not only to traditional elements such as biological samples and genetic information, but also to health records and behavioral data generated through the use of new technologies and new products.	Measures for the Ethical Review of Life Sciences and Medical Research Involving Humans (2023)
Electronic/remote informed consent Electronic signature Drug Direct to Patient (DTP)	National Medical Products Administration. (NMPA) Standing Committee of the National People’s Congress	Electronic informed consent may be considered for clinical trials. Ensure that the process meets regulatory requirements. Electronic Signature requirement.	The guidance for the application of decentralized clinical trials in clinical development of rare disease drugs (2024) Guideline for Patient-Centered Clinical Trial Design (Interim, 2023) Guideline for Patient-Centered Clinical Trial Conduct (Interim, 2023) Guideline for Patient-Centered Benefit–Risk Assessment (Interim, 2023) Electronic Signature Law of the People’s Republic of China (2019)
Privacy protection	Cyberspace Administration of China (CAC)	Emphasizes data privacy and security in clinical research. Ensures that all patient data is managed according to this law, including obtaining explicit consent for data collection, ensuring secure storage, and restricting access to data.	Personal Information Protection Law (2021)
Data cybersecurity	Cyberspace Administration of China (CAC)	Ensures that any data used in clinical trials, particularly remote monitoring data or data from wearable devices, is handled in accordance with security protocols to prevent breaches or misuse. Requirements for data localization and cross-border data transfer, which may be a concern for multinational trials.	Data Security Law (2021)

**Table 7:** The elements of decentralized clinical trials from the FDA, EMA, and NMPA.

FDA (USA)	EMA (EU)	NMPA (China)
Specific plans include the use of local health care facilities, visits, direct distribution of the IP, etc., and the feasibility, design, and implementation.	The rights, safety, dignity, and wellbeing of the participants.	Compliance with the current legislation system, like GCP, local ethical and data regulations.
Discussed early with the relevant FDA review divisions.	Compliance with the current legislation system, such as GCP and GDPR.	Fit-for-Purpose Patient centered
Up-front risk assessment and management, like data	Design, development, and implementation.	Risk/quality management plan
A physical location where could be inspection	A formal written document described the decentralized elements planned in the clinical trial.	Design and implementation include DCT elements, key data, facilities, digital endpoint, training, etc.
DCT Design	Risk–benefit assessment of decentralized elements	Stakeholder responsibility and communication
Remote Visits and Trial-Related Activities	Medical devices, digital technology, and electronic data.	Data security and compliance
Digital Health Technologies (guidance)	Roles and responsibilities	Source data
Roles and Responsibilities	INFORMED CONSENT PROCESS	Regulatory authority communication
Informed Consent and IRB Oversight	Delivery of IMP and administration at home.	Remote recruitment
determination of the appropriateness of investigational products (IP) for use in a DCT	Trial-related procedure at home.	Patient interaction/education
Packaging and Shipping of IP	Data collection and management, source data.	e-Inform Consent
Safety Monitoring Plan	Trial monitoring	Remote visit
Software Used in Conducting DCTs		IMP direct to Patient/IMP Home supply
		Digital Health Technologies
		Remote assessment/eCOA
		Nearby medical resources (qualification/education)
		Safety monitoring

In addition to the similarities, the differences are reflected in the following areas: First, while the Chinese regulatory authority has emphasized the immense potential of digital technologies in new drug development, its first comprehensive regulatory guidance on DCTs, launched in 2024, primarily focuses on rare diseases. This may reflect a cautious stance by Chinese regulatory authorities, favoring a pilot program within narrowly defined populations. Moreover, the United States has developed a systematic approach to supporting the key enablers of DCTs, particularly digital health technologies (DHTs). The FDA has introduced DHT-related concepts and launched a series of initiatives, such as Software as a Medical Device (SaMD), which broadens the traditional definition of medical devices, and the Pre-Cert Pilot Program. Finally, Europe has placed greater emphasis on empowering patients through education and training, enabling them to actively participate in medical research and development. Initiatives such as the European Patients’ Academy on Therapeutic Innovation (EUPATI), launched in 2012, aim to enhance effective patient–researcher partnerships and foster global health innovation (35). Moreover, the Innovative Medicines Initiative (IMI)—a public–private partnership co-funded by the European Union and EFPIA member companies—has focused on developing advanced technologies and methodologies to improve drug development. By advancing better biomarkers and clinical trial designs, IMI has helped bridge gaps between academia, industry, and regulators and has accelerated research in personalized medicine and digital health technologies (25).

In summary, while regional regulatory expectations for DCTs exhibit certain differences, their commonalities far outweigh the divergences. This convergence likely reflects the broader trend of globalization and sustained international dialogue over the past several decades. Given its central role in promoting global regulatory alignment, the International Council for Harmonization (ICH) should take a proactive role in leading further harmonization efforts related to DCTs, ensuring consistency and efficiency across regions.

## Discussion

This article summarizes the current progress made by academia, think tanks, and regulatory bodies in exploring the opportunities and challenges of DCTs. These topics can be categorized into four dimensions, highlighting eight themes. The first dimension relates to the new paradigm introduced by innovative technologies, which also represents the most immediate and visible impact. Within this paradigm, challenges such as fairness and accessibility for trial participants must be prioritized. The third dimension relates to governance and public trust within this paradigm shift. Cybersecurity, privacy protection, and the ethical implications of emerging technologies all play a critical role in ensuring the sustainability of this model, as public trust is a prerequisite for long-term development. The final dimension, which becomes critical once the previous themes have been addressed, concerns infrastructure. Reliable Internet connectivity, direct-to-patient drug distribution systems, and robust telemedicine capabilities constitute the foundation for the sustainable development of DCTs.

In addition, this article examines the current regulatory expectations and frameworks. Major regulatory authorities have accumulated considerable practical experience and have begun to establish preliminary regulatory structures. However, two points deserve further consideration: First, DCTs are not simply a digital extension of traditional clinical trials. Second, despite the issuance of various DCT-related guidelines by major regulatory agencies, the current regulatory expectations and frameworks may still exhibit some degree of fragmentation. Modules such as digital health technology, patient participation, and education are beginning to shape an innovative regulatory system. Furthermore, the regulatory maturity of DCTs varies significantly across regions influenced by differing policy priorities: the U. S. emphasizes efficiency, Europe focuses on equity, and China prioritizes stability. The U. S. FDA has demonstrated relatively advanced regulatory action, issuing specific guidance on DCTs (e.g., the 2023 draft) and launching initiatives such as the Pre-Cert Program and the Software as a SaMD framework. In contrast, EMA relies heavily on coordination among national competent authorities, often resulting in fragmented and uneven implementation across member states. China’s regulatory framework for DCTs continues to evolve, with ongoing efforts to address infrastructure-related challenges, such as regional disparities in capacity and the gradual adoption of electronic consent (eConsent). Although the COVID-19 pandemic catalyzed the global adoption of DCTs, regulatory harmonization remains challenging due to differences in terminology, infrastructure gaps, and a fragmented legal and governance landscape that hinders cross-border trial integration. This fragmented governance of DCTs across regions affects both sponsors and patients. Sponsors face the need to reassess compliance costs and financial return uncertainties arising from inconsistent regulations, and patients may have limited access to DCTs due to different local rules on telemedicine, eConsent, and home healthcare. Finally, lessons from DCT pilot programs show some key challenges. For example, according to the FDA’s final 2022 report on the Pre-Cert Program, legislative changes are necessary to implement this pilot program (36). Meanwhile, pharmaceutical companies—especially smaller ones—still struggle with unclear regulatory paths, which hinders broader adoption and innovation of DCTs. Therefore, compared to the relatively mature frameworks governing traditional clinical trials, this emerging system for DCTs still has a long way to go.

In this regard, Canada’s Clinical Trials Remote Access Framework (CRAFT) and CTTI offer two distinct and valuable perspectives on regulatory approaches to DCTs. CRAFT has published a paper outlining a distinct set of considerations for DCTs (37). Their framework includes eight key elements: (a) Infrastructure, personnel, and system development; (b) Costs and funding requirements; (c) Trial planning and conduct; (d) Health Canada regulations, guidelines, and inspections; (e) Ethics review; (f) Patient privacy; (g) Trial agreements, indemnity, and insurance; (h) Engagement, communication, and advocacy. As previously mentioned, another notable CTTI established in 2007 through a collaboration between Duke University and the U. S. Food and Drug Administration focuses on advancing digital health trials and has identified six key areas of emphasis: developing novel endpoints, planning decentralized trials, selecting and testing a digital health technology, managing data, supporting sites, and interacting with regulators (38). CRAFT integrates infrastructure, personnel, funding, regulatory guidance, ethics, patient privacy, indemnity, and communications into a cohesive framework. They place greater emphasis on practical infrastructure and system-level coordination beyond purely regulatory requirements. In contrast, CTTI’s six focus areas offer a structured and innovation-driven approach to designing and conducting digital and decentralized trials, distinguishing it from traditional regulatory guidance.

Overall, the literature shows growing support for DCTs. However, digital technology alone is not enough to unlock their full potential. Cooperation among regulatory agencies, industry, patients, and others is also crucial. Progress depends on engaging all stakeholders, establishing unified regulatory coordination, and building a robust ecosystem that fills operational gaps and improves regulatory science to support innovation and sustainability.
